# Intravenous iron is non-inferior to oral iron regarding cell growth and iron metabolism in colorectal cancer associated with iron-deficiency anaemia

**DOI:** 10.1038/s41598-021-93155-2

**Published:** 2021-07-01

**Authors:** Hafid O. Al-Hassi, Oliver Ng, Rayko Evstatiev, Manel Mangalika, Natalie Worton, Manuela Jambrich, Vineeta Khare, Oliver Phipps, Barrie Keeler, Christoph Gasche, Austin G. Acheson, Matthew J. Brookes

**Affiliations:** 1 Research Institute in Healthcare Science, Faculty of Science and Engineering, University of Wolverhampton, Wolverhampton, UK; 2 NIHR Nottingham Biomedical Research Centre and the University of Nottingham, Nottingham, UK; 3 Department of Internal Medicine III, Division of Gastroenterology and Hepatology, Medical University of Vienna, Vienna, Austria; 4 The Royal Wolverhampton NHS Trust, Wolverhampton, UK

**Keywords:** Gastrointestinal cancer, Cancer, Cell biology, Gastroenterology

## Abstract

Oral iron promotes intestinal tumourigenesis in animal models. In humans, expression of iron transport proteins are altered in colorectal cancer. This study examined whether the route of iron therapy alters iron transport and tumour growth. Colorectal adenocarcinoma patients with pre-operative iron deficiency anaemia received oral ferrous sulphate (n = 15), or intravenous ferric carboxymaltose (n = 15). Paired (normal and tumour tissues) samples were compared for expression of iron loading, iron transporters, proliferation, apoptosis and Wnt signalling using immunohistochemistry and RT-PCR. Iron loading was increased in tumour and distributed to the stroma in intravenous treatment and to the epithelium in oral treatment. Protein and mRNA expression of proliferation and iron transporters were increased in tumours compared to normal tissues but there were no significant differences between the treatment groups. However, intravenous iron treatment reduced ferritin mRNA levels in tumours and replenished body iron stores. Iron distribution to non-epithelial cells in intravenous iron suggests that iron is less bioavailable to tumour cells. Therefore, intravenous iron may be a better option in the treatment of colorectal cancer patients with iron deficiency anaemia due to its efficiency in replenishing iron levels while its effect on proliferation and iron metabolism is similar to that of oral iron treatment.

## Introduction

Iron is a vital element for many biological functions including oxygen delivery, metabolism, growth and DNA synthesis^1,2^. However, excess in luminal iron can create reactive oxygen species which can induce mutation of the mismatch repair genes and subsequently leads to microsatellite instability (MSI) causing DNA damage and carcinogenesis^3^. Cellular absorption of dietary non-haem iron occurs in the duodenum and upper jejunum via the duodenal cytochrome b-like ferrireductase (DcytB)^4^ and imported into the cell by the divalent metal transport 1 (DMT1). However, these cellular iron transporters are now also known to be present within the colonic epithelium^5^ and are modified in bowel cancer^6^.

After absorption iron is either stored as ferritin or exported from cells via the basolateral ferroportin (FPN)^7,8^ facilitated by the membrane protein hephaestin (HEPH) or plasma protein ceruloplasmin. Iron can then be transported in the extracellular fluid and plasma, bound to transferrin^9^. Cells then obtain iron via binding of the iron-transferrin complex to transferrin-receptor 1 (TfR1)^10,11^ and released from the endosome via DMT1 to again form a labile iron pool, which can be taken up for the cellular processes^12^.

Cellular iron transporter levels are controlled at a post-transcriptional level by iron-responsive binding proteins (IRP) 1 and 2^13^. When activated by iron-deficiency, IRPs bind to iron-responsive elements (IREs) in the untranslated regions of messenger RNA including TfR1 and ferritin and promote the translation of TfR1 and repression of ferritin which increases the labile intracellular iron pool with decreases in iron export, utilisation and storage^14,15^.

Colorectal cancer (CRC) is a major cause of cancer-related mortality worldwide^16^ and associated with iron deficiency anaemia (IDA). Oral iron (OI) treatment of anaemia may prove inappropriate because colonic iron is implicated in gut mucosal inflammation, CRC growth, stimulation of oxidative stress and reduction of antioxidant vitamins^17–21^. Studies in animal models showed that high dietary iron induced intestinal inflammatory responses, impaired intestinal immune and barrier function^22^ and CRC growth in mice in the presence of the colonotropic carcinogen, azoxymethane^23^. In addition, it has been shown that enteral iron supplementation promotes CRC progression in animal models with *APC* gene mutation^24^.

However, most studies on the association between dietary iron intake and development of CRC were conducted in animal models or on cell lines using supra-physiological doses of iron. One cohort study found no association between dietary iron and the risk of CRC in women^25^. On the other hand, systemic iron replacement does not increase carcinogenesis despite adequately replenishing iron stores with high profile of safety and tolerance^26,27^. However, in humans the consequences of elevated luminal or systemic iron on CRC still not well understood^28^. We hypothesize that excess in luminal iron due to OI therapy has the potential to favour tumour development and proliferation whereas IV iron treatment does not have this effect.

We have previously demonstrated that intravenous iron therapy (IV) is more effective in the treatment of clinical anaemia compared with OI treatment^29^. Based on these clinical findings, this study was designed to examine the route of iron therapy on mucosal iron distribution and evaluate its cellular and molecular effects on tumour cells.

## Results

### Clinical outcome

**Figure 1:**
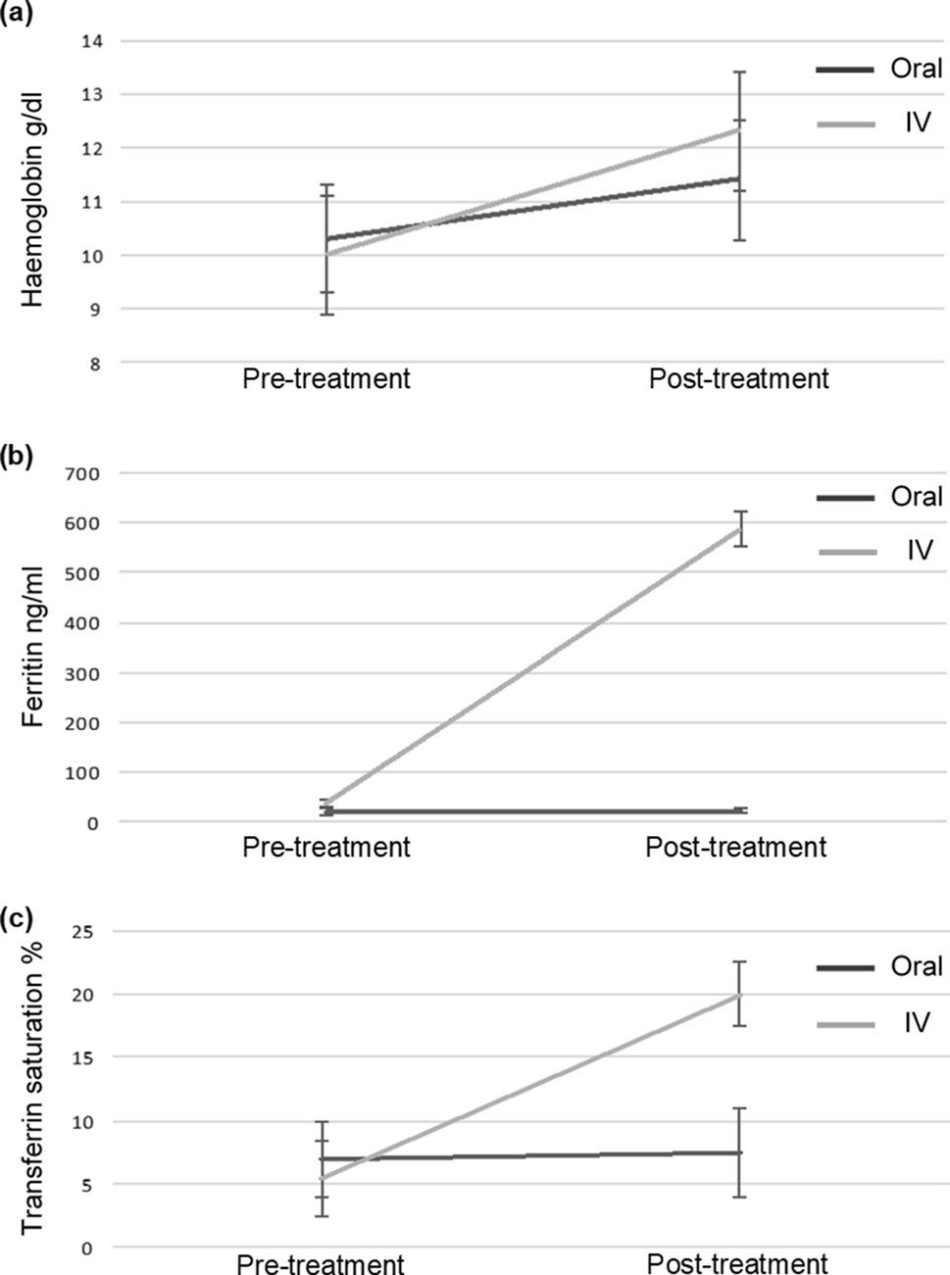
IV treatment promotes ferritin levels and saturation of haemoglobin and transferrin; (**a**) Haemoglobin (**b**) Ferritin (**c**) Transferrin.

All patients reported compliance with OI therapy. Patients were similar at recruitment for age, sex, Dukes stage, haemoglobin, ferritin and transferrin saturations. Ferritin levels were significantly higher in the IV group (median ferritin 588 ng/mL IV versus 22 ng/mL oral, p = 0.001). Saturation levels of haemoglobin and transferrin were also higher in the IV group by day of surgery (Table 1). Patients in the OI group remained iron deficient, ferritin 22 ng/mL. No patients had a pre-operative transfusion (Fig. 1).

**Table 1:** Clinical outcome.

	Oral iron (n = 15)	IV iron (n = 15)	p
Age (years)^a^	74 (46–82)	74 (53–85)	0.662
Sex ratio (M:F)	10:5	8:7	0.758
**Haemoglobin (g/dL)**^**b**^			
Recruitment	10.3 (1.0)	10.0 (1.7)	0.616
Day of surgery	11.4 (1.1)	12.3 (2.1)	0.131
**Ferritin (ng/mL)**^**c**^			
Recruitment	21 (14–45)	39 (12–204)	0.384
Day of surgery	22 (16–51)	588 (318–1415)	0.001*
**Transferrin saturations (%)**^**c**^			
Recruitment	7 (4–16)	5.5 (2–14)	0.301
Day of surgery	7.5 (4–13)	20 (17–24)	0.290
**Dukes n (%)**			
A	0	2 (13.3)	0.845
B	9 (60)	10 (66.7)	
C	6 (40)	3 (20)	
**Site n (%)**			
Caecum	6 (40)	9 (60)	0.938
Ascending colon	0	0	
Hepatic flexure	1 (6.7)	1 (6.7)	
Transverse colon	3 (20)	0	
Splenic flexure	2 (13.3)	1 (6.7)	
Descending colon	0	1 (6.7)	
Sigmoid	2 (13.3)	0	
Rectum	1 (6.7)	3 (20)	
Iron therapy	Ferrous sulphate 200 mg BD PO	Ferric carboxymaltose 1000 mg Single dose IV	–
Days before surgery^a^	25 (16–36)	26 (15–34)	0.798
Transfusions	0	0	–

*p < 0.05.

^a^Median (range).

^b^Mean (Standard deviation).

^c^Median (Interquartile range).

### Microsatellite instability

MSI was present in 23% of tumours, with five cancers demonstrating loss of MLH1 and two cancers demonstrating loss of both MSH2 and MLH1. Of these seven tumours, four MSI tumours were in the OI group and the remaining three in the IV group. MSS and MSI tumours were compared for all RT-PCR and immunohistochemistry outcomes and across treatment groups (Supplementary Tables S1 and 2). Sub-analyses are discussed below.

### Cellular proliferation and Wnt signalling

No significant differences between tumours in oral and IV groups were seen. Exclusion of MSI phenotype did not alter results between treatment groups, (Supplementary Tables S1 and 2).

**Figure 2:**
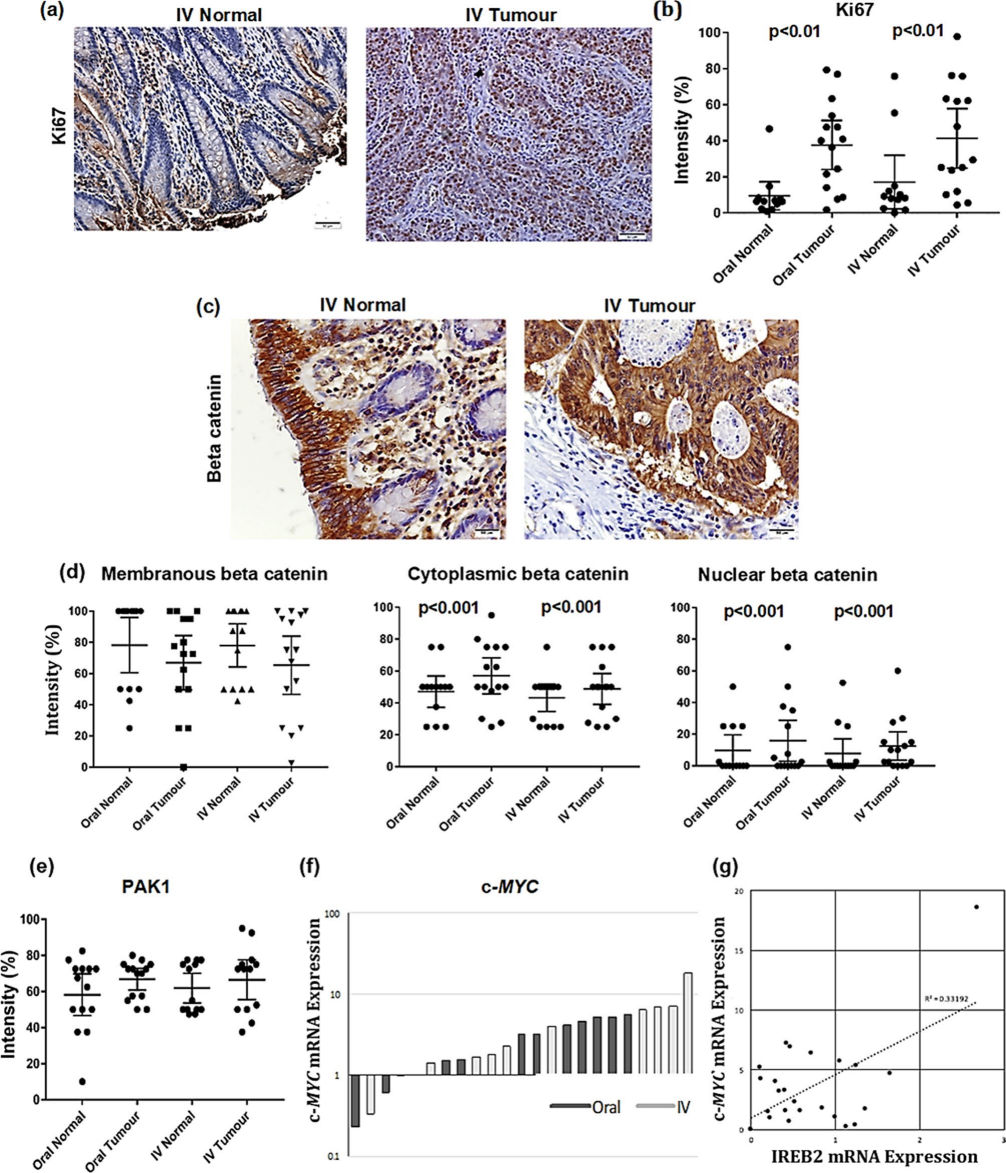
IV treatment did not induce changes in proliferation or Wnt signaling pathway over oral iron treatment (**a**) Ki67 immunostaining; (**b**) analysis for PKi67 protein, dot plots with mean and standard deviation; (**c**) immunostaining of beta-catenin in normal and Tumour tissues; (**d**) Dot plot analysis with mean and standard deviation of membranous, cytoplasmic and nuclear beta catenin expression; (**e**) Dot plot analysis with mean and standard deviation of PAK1 immunostaining; (**f**) Real-time PCR fold change in c*-MYC* gene expression in tumour tissues with respect to normal tissue; (**g**) IREB2 correlation with c*-MYC* scatter plots with regression line.

The proliferation marker Ki67 immune staining was mainly nuclear with low immuno-reactivity in normal tissue but significantly higher in tumours (p < 0.01) and no difference was seen between the treatment groups. (Fig. 2a,b).

Membranous and cytoplasmic β-Catenin, the main intracellular signal transducer in the Wnt signalling pathway, had immunoreactivity in both normal and tumour tissues. However, nuclear staining was only seen in some tumour tissues and no normal tissues (Fig. 2c).

There were no significant differences in β-Catenin membranous expression between normal and tumour tissues. In contrast, both cytoplasmic and nuclear immunoreactivity for β-Catenin showed significant differences between normal and tumour tissue (p < 0.001) and no differences were seen between treatment groups (Fig. 2d). The p21-activated kinase 1 (PAK1), a downstream effector of GTPases overexpressed in many tumours, showed cytoplasmic immunoreactivity in all tissues, both normal and tumour but there were no significant differences between normal and tumour or between treatment groups (Fig. 2e).

Wnt signalling target gene c*-MYC* mRNA fold-changes were significantly higher in tumour cells compared with normal (p < 0.001) but did not significantly differ between treatment groups, although with a smaller mean fold-change in intravenous 3.2 (1.90–4.50 95% CI) versus oral 4.7 (4.50–8.20 95% CI) (Fig. 2f). IRP2 mRNA levels positively correlated with MYC mRNA levels (R2 = 39%, p < 0.01) (Fig. 2g).

### DNA damage and apoptosis

**Figure 3:**
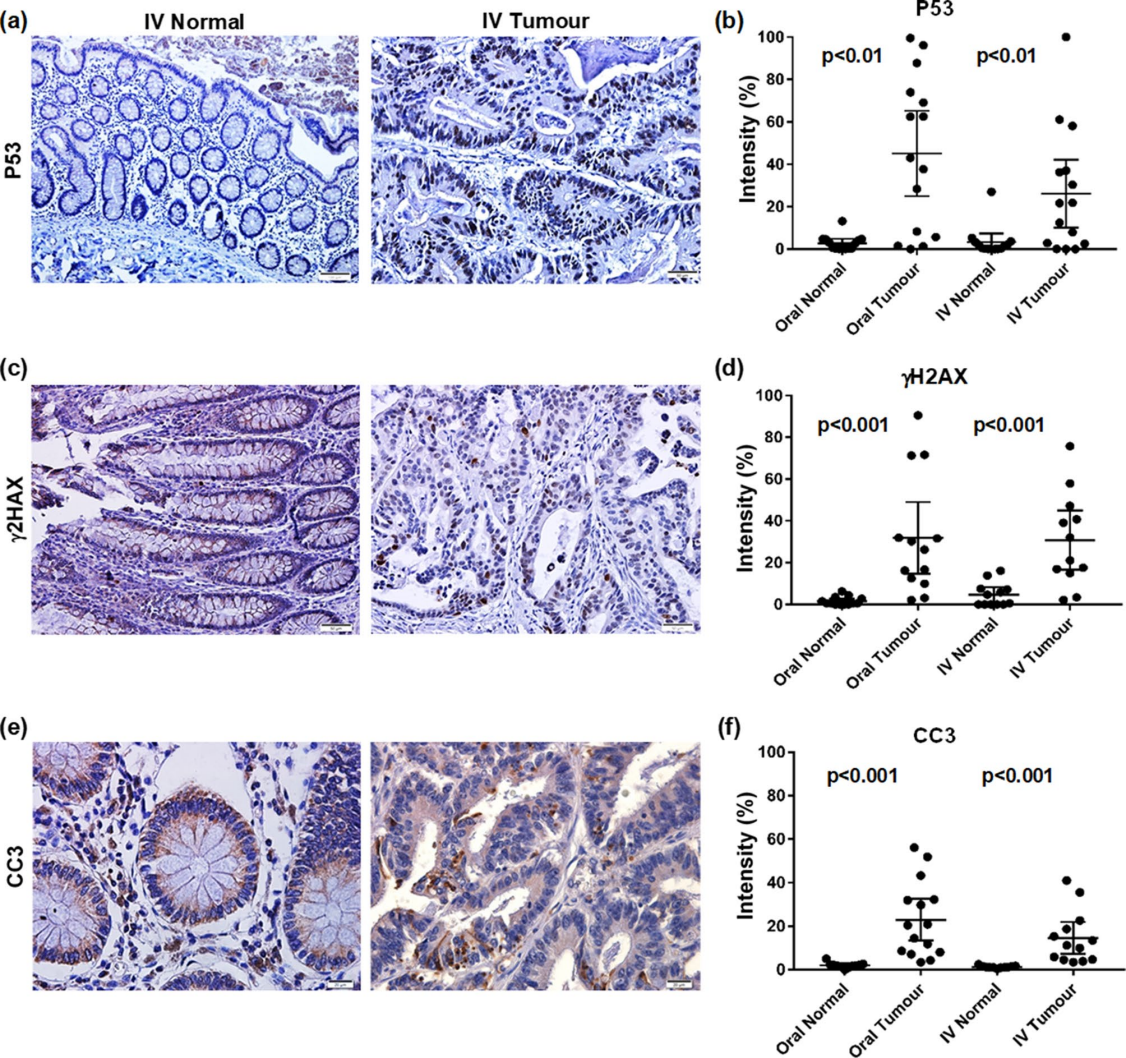
Treatment with IV did not induce significant changes in apoptosis or DNA damage in normal and tumour tissues compared with oral iron treatment. (**a**) P53 immunostaining in normal and Tumour tissues; (**b**) analysis for P53 protein, dot plots with mean and standard deviation; (**c**) γH2AX immunostaining in normal and Tumour tissues; (**d**) analysis for γH2AX protein, dot plots with mean and standard deviation; (**e**) CC3 immunostaining in normal and Tumour tissues; (**f**) analysis for CC3 protein, dot plots with mean and standard deviation.

The tumour suppressor gene p53 revealed no positive immunoreactivity in the nuclei in 89% of normal tissue. In comparison, only 20% of tumours had no positive nuclei (Fig. 3a) and there was a statistically significant difference in immunoreactivity between normal and tumour tissue (p < 0.01) (Fig. 3b). However, there were no significant differences in p53 expression between treatment groups. MSI tumours were associated with higher p53 expression compared to the MSS group (p = 0.01) (Additional file 5). DNA double strand breaks, detected through γH2AX protein staining showed no or low nuclear immuno-reactivity in all normal tissue. Tumour, as expected, had higher immuno-reactivity (Fig. 3c). There was a statistically significant difference between normal and tumour tissues (p < 0.001) (Fig. 3d). No difference was seen between treatment groups in normal or tumour tissue. There was almost no positive staining for the apoptosis marker cleaved caspase 3 (CC3) protein in normal tissue. However, all tumours except one had some CC3 immunoreactivity (Fig. 3e) which was statistically significant compared to that of normal tissues (p < 0.001) (Fig. 3f). There were no differences between treatment groups.

### Tissue iron loading and storage

**Figure 4:**
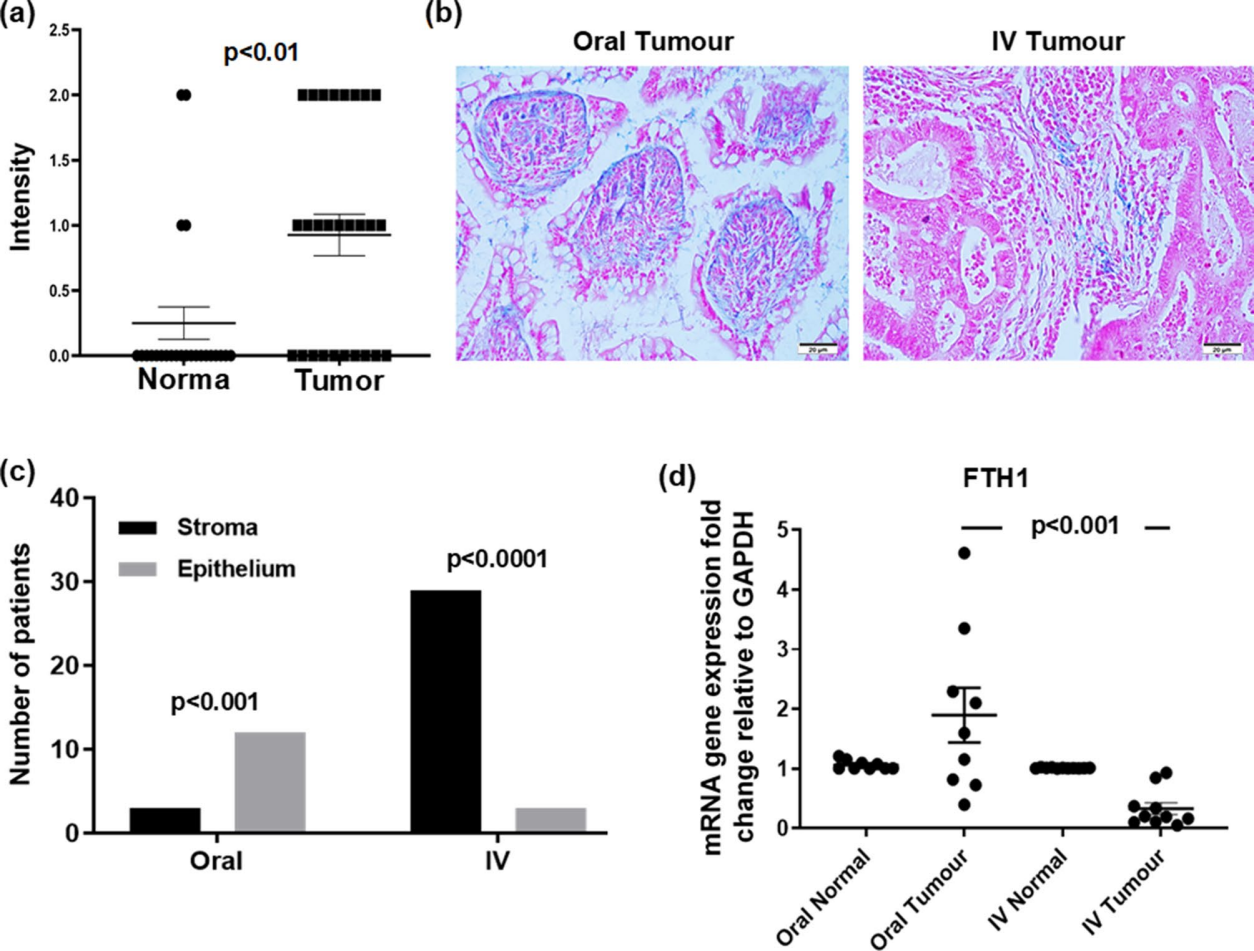
Perl’s Prussian blue staining was distributed to the stroma in IV treated patients while its expression was mainly in the tumour epithelium of oral iron treated patient and FTH1 has significantly reduced in tumours with IV treatment. (**a**) Positive staining in normal and Tumour cells; (**b**) analysis for Perl’s Prussian blue, dot plots with mean and standard deviation; (**c**) Chi square analysis shown expression of Perl’s Prussian blue in Tumour and stroma of Tumour tissue from patients treated with oral or IV iron. (**d**) Real-time PCR fold change in FTH1 gene expression comparing oral versus intravenous iron groups.

Iron loading was significantly increased in tumour tissues compared to normal tissues regardless of the treatment group (p < 0.01) (Fig. 4a). In tumour tissues, Perl’s Prussian blue expression was significantly higher in the epithelial cells from the oral group compared with the IV group (p < 0.01) whereas in the IV group it was significantly distributed to the stroma (p < 0.0001) (Fig. 4b,c). *FTH1* mRNA that encodes the heavy subunit of ferritin, was reduced in tumours compared to normal tissues with a greater reduction in the IV group, (p < 0.001) (Fig. 4d) (Supplementary Table S3).

#### Cellular iron transport and iron regulation

**Figure 5:**
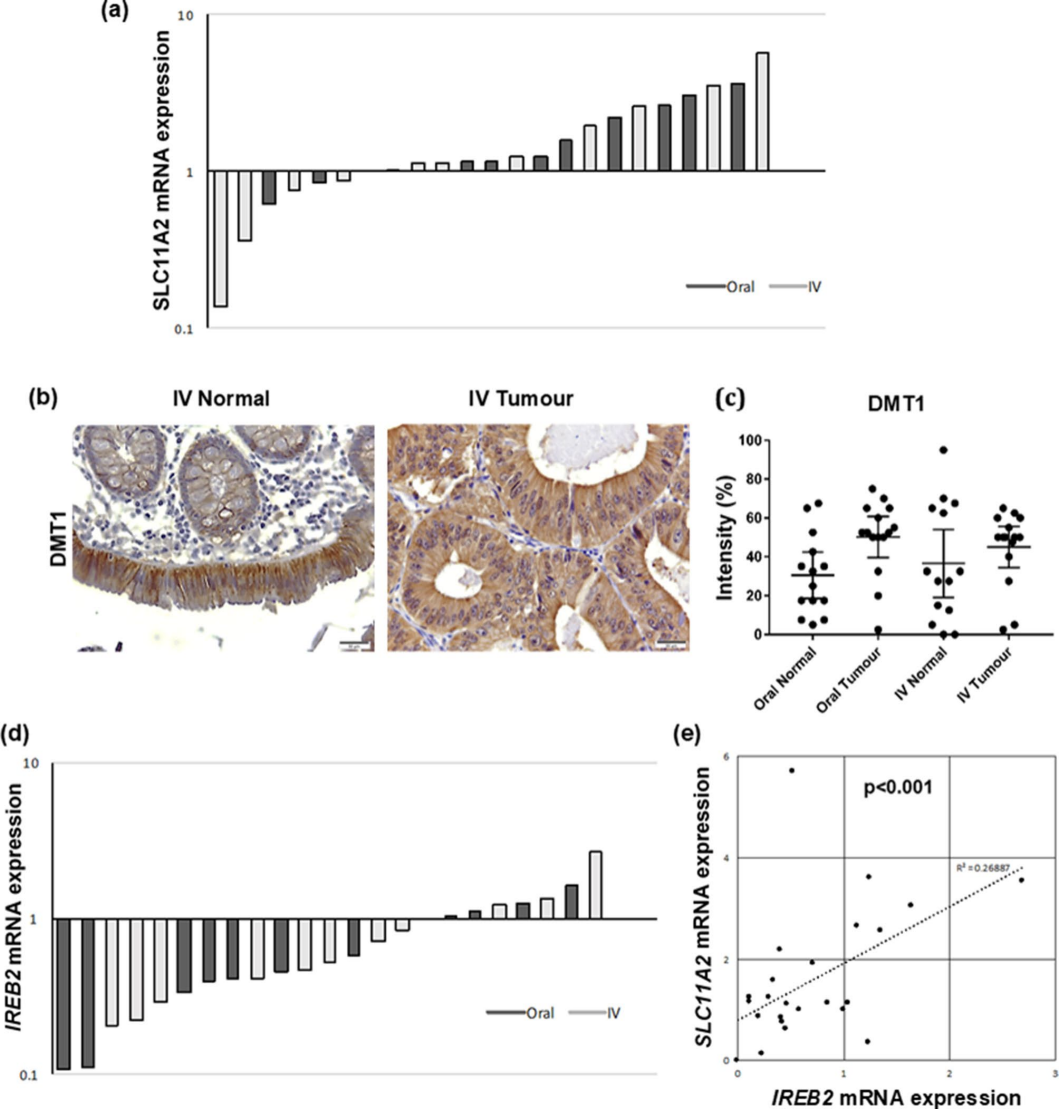
IV iron treatment did not affect expression of iron metabolism genes significantly in normal or tumour tissues compared with oral iron treatment. (**a**) Real-time PCR fold change in SLC11A2 (DMT1) gene expression comparing oral versus intravenous iron groups. (**b**) Immunostaining of DMT1 in normal and Tumour tissues. (**c**) Analysis for DMT1 protein, dot plots with mean and standard deviation. (**d**) Real-time PCR fold change in IREB2 gene expression comparing oral versus intravenous iron groups. (**e**) IREB correlation with DMT.

The iron transport gene *SLC11A2* expression in OI versus IV and the immunoreactivity of its encoded protein DMT1 were higher in tumours compared to normal tissues but this increase did not reach statistical significance (p < 0.07) (Fig. 5a–c, respectively). In OI compared to IV, RT-PCR showed IRP2 (*IREB2*) was reduced (Fig. 5d) and IRP2 mRNA levels positively correlated with *SLC11A2* (R^2^ = 67%, p < 0.001, Fig. 5e).

**Figure 6:**
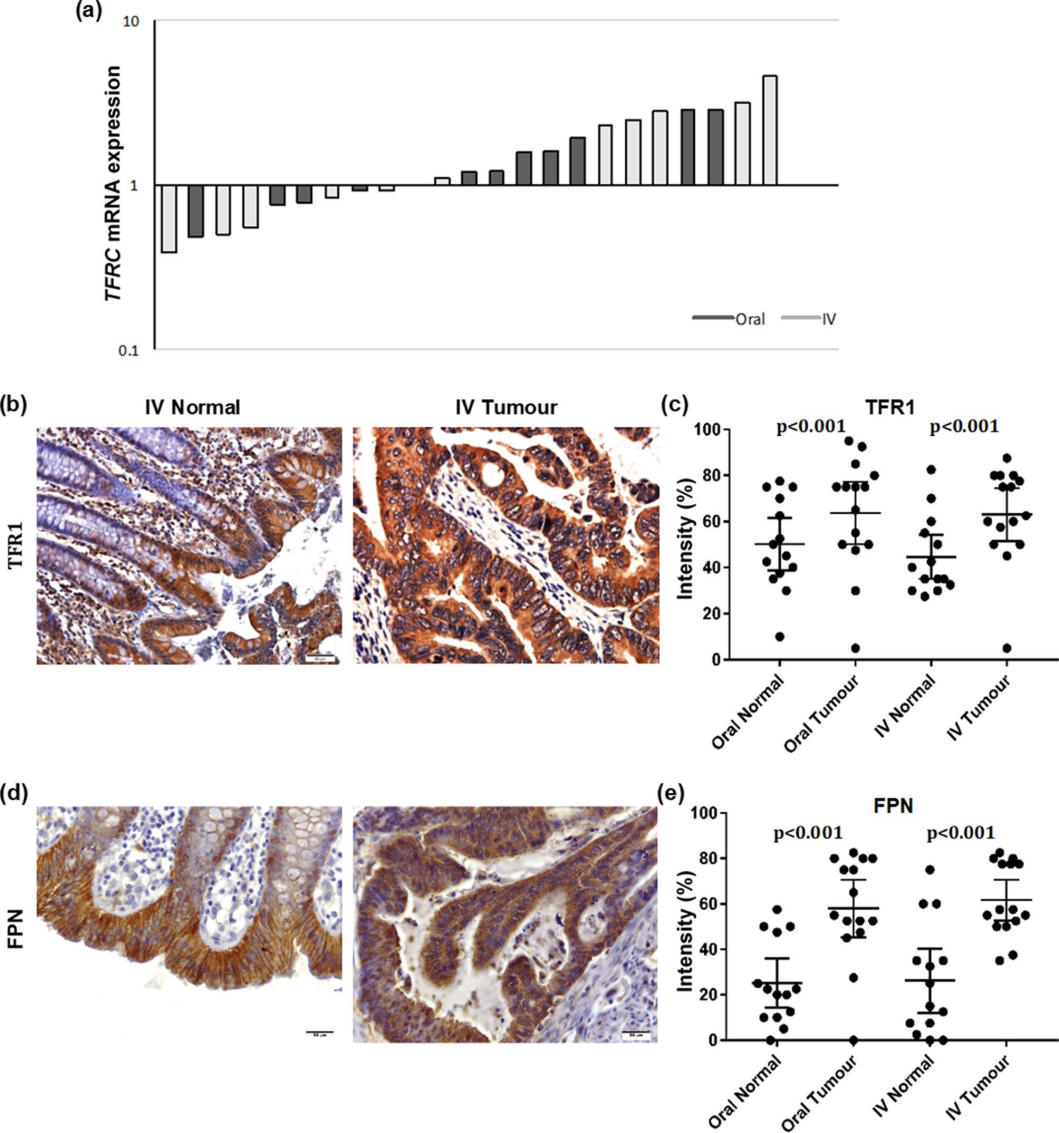
Expression of iron metabolism proteins did not significantly differ when patients treated with oral or IV iron (**a**) Real-time PCR fold change in *TFRC* (TFR1) gene expression comparing oral versus intravenous iron groups. (**b**) Immunostaining of TFR-1 in normal and Tumour tissues. (**c**) Analysis for TfR1 protein, dot plots with mean and standard deviation. (**d**) Immunostaining of ferroportin in normal and Tumour tissues (**e**) analysis for ferroportin protein, dot plots with mean and standard deviation.

*TFRC* gene expression in OI treatment was not significantly different to that in IV treatment group (Fig. 6a). However, the immunoreactivity of its protein TfR1 was significantly higher in tumour tissues compared with normal tissues (p < 0.0001) (Fig. 6b,c respectively). Furthermore, immune-reactivity of ferroportin was significantly higher in tumour compared to normal tissue (p < 0.001) (Fig. 6d,e respectively). Staining for DMT1, TfR1 and ferroportin was altered in tumours and localised to the cytoplasm whereas in normal tissues their expression was membranous (Figs. 5b, 6b,d, respectively). No significant differences in the iron transporters were seen between treatment groups and sub-analysis of microsatellite instability did not alter these results.

## Discussion

This study examined for the first time in humans, two groups of anaemic patients with CRC, randomised to oral or IV iron therapy. It compared molecular changes between normal and tumour tissue and between treatment groups. Although, the overall iron dosages (oral or IV) are comparable, IV iron therapy successfully replenished body iron whereas the OI group remained iron deficient. However, caution should be considered when interpreting the efficacy of these agents as they are likely to have different bio-availabilities.

Despite increase in body iron following IV treatment, the effects of oral and IV iron treatments were similar in terms of iron metabolism, proliferation and apoptosis levels in tumours and paired normal tissues. The lack of significant differences between the treatments may suggest that IV iron treatment does not worsen or exacerbate the disease. Studies in mouse models of colitis-associated CRC by contrast, showed that OI increases the number and size of tumours when compared to an iron-deficient diet and IV^26,30^. However, this model of inflammatory colorectal carcinogenesis is unlike most sporadic CRC in humans. Another consideration is the dietary iron intake, in Seril et al. study, mice were fed iron-deficient diet followed by supplementation of high supra-physiological doses of iron over a relatively longer time period when compared to the time period over which carcinogenesis occurs in humans^26^. On the contrary, our human participants were still consuming a normal Western diet commonly replete in dietary iron, with an average daily iron consumption of 15–20 mg and body absorption of 1–2 mg/day. Furthermore, the patients were treated with either oral ferrous sulphate 200 mg tablet twice a day for two weeks or intravenous ferric carboxymaltose 1000 mg per week with a maximum does of 2000 mg according to the patient bodyweight and inclusion haemoglobin value^29^. Thus, results on the effects of OI using animal models and cell lines, should be extrapolated to humans with caution.

This study has demonstrated for the first time that the route of iron administration affects the localisation of iron loading; iron was localised to the stromal tissues in the IV treatment group. The implications of this are uncertain, however, OI supplementation results in an excess of biologically available iron to the colonic mucosa^31^. Excess of luminal iron causes downregulation of DcytB and FPN1 mRNA and protein expression leading to ‘mucosal block’ which is a suppression of iron absorption^32,33^. Assuming that not all the iron passing through the gastrointestinal tract was absorbed in the OI treated patients, this may indicate that the excess in luminal iron is accessible to the tumour and that less iron is bioavailable to tumour cells from patients treated with IV iron treatment. Hence, keeping iron compartmentalised away from the tumour may be a beneficial effect of using IV iron treatment in CRC patients with IDA. Further, differential compartmentalisation and the tumour microenvironment could all potentially influence intracellular tumour iron loading, macrophage and immune function. However, a larger study is warranted to confirm these findings. Furthermore, differential compartmentalisation of iron within tissue should also be interpreted carefully. Haemosiderin stains intensely with Prussian blue and ferritin only at high concentrations^34^. Haemosiderin may be largely inert and biologically inactive, reflecting instead a secondary mechanism for iron storage when ferritin storage is exceeded^35^. The biologically active labile iron pool however is not seen or quantified with Prussian blue and has instead been inferred. It is remained to be elucidated whether the formulations of the OI and IV iron treatments can also affect the outcome.

Iron importers TfR1 and DMT1 at both mRNA and protein level were increased in tumours, with no differences seen between treatment groups. Ferritin heavy chain mRNA was also reduced. The net effect of decreased iron storage and increased iron import would be an increase in the labile iron pool. This appears to be occurring due to a change of normal iron sensing mechanisms. IRP2 was decreased in tumours, a normal response to high intracellular iron, but this did not lead to a reduction in TfR1 or an increase in FTH1 as expected. In fact, TfR1 expression increased and FTH1 mRNA expression decreased with no correlation with IRP2 mRNA expression. This is contrary to findings by Horniblow in which IRP2 and TfR1 expression both increased in CRC and correlated with each other^36^.

This effect had previously been demonstrated in relationship to *APC* wild type cancer cell lines, whereby the regulation of iron stores appeared to be IRE/IRP dependent with normal iron decreasing IRP2 with subsequent decreases in TFR1 and DMT1^24^. In cancers with a mutation in *APC*, the regulation of colon cancer cells iron stores became IRE/IRP independent and despite high iron, TFR1 and DMT1 expression increased. This could be reversed when APC was transfected into these cells^24^. This model would hypothesise that IRE/IRP sensing might be bypassed by beta-catenin TCF signalling and overwhelmed by huge increases in iron. In this study, there was no difference in iron regulation between MSI and MSS (and likely APC pathway) tumours.

The reduction in FTH1 expression observed in the IV treatment group may promote immune evasion by tumours. This is because tumours release FTH1 to induce an immunosuppressive microenvironment by re-programming dendritic cells so they induce production of the anti-inflammatory cytokine IL-10 from regulatory T cells, hence, allowing the tumour to escape immune surveillance^37^. On the other hand, reduction in FTH1 may lead to the accumulation of free iron and subsequent increase in ROS which in turn can promote tumour metastasis and immune suppression^38,39^.

Changes in iron metabolism were also not related to c*-MYC* expression, which correlated with IRP2 but showed no relationship with iron transport (*SLC11A2* and *TFRC*) or storage (FTH1). Previous in vitro experiments examining transcriptional targets of c*-MYC* have yielded mixed results. One study has shown increased c*-MYC* causes an overexpression of IRP2 and a reduction of FTH1 but had no effect on *TFRC*^40^. Others have shown that c*-MYC* can independently induce TfR1^38^ and that transfection of c*-MYC* to colon cancer cell lines increases ferritin heavy chain transcription^41^. Iron export via ferroportin was also altered in tumours with increased expression and mis-localisation from the membrane to the cytoplasm, the latter potentially reducing iron export from cells again increasing labile iron.

The strength of this study is that, to the best of our knowledge, this is the first time it has been shown that intravenous and oral treatments of iron deficiency anaemia do not differ with regards to cellular iron metabolism, cell proliferation and apoptosis, suggesting, biological non-inferiority of IV to OI treatment.

Limitations to this study include the likelihood of marked heterogeneity in the tumours and treatment despite matching for tumour stage, histology and sub-analysis by MSI status. Also, heme iron pathways play a smaller but significant role in iron absorption and are neither controlled for nor examined in this study. Furthermore, the small window of intervention, just over two weeks, may also be insufficient to alter the biology of the tumour. Nevertheless, this short period of exposure reflects the real-life use of iron replacement therapy in patients with CRC. However, in view of these preliminary findings, further investigations are warranted to confirm the efficacy of IV iron therapy in CRC patients without promoting tumour progression. In order to understand the impact of parenteral iron on the mucosal and tumour microenvironment, future in vitro and in vivo studies can be performed using models such as co-culture or organoid culture systems. Future trials should also include food diaries to estimate the amount of iron intake by the participants.

In conclusion, although IV treatment is more effective in correcting iron deficiency and clinical anaemia despite iron avid tumours^29,42–44^, our data did not fully support our hypothesis. However, IV iron is not only biologically non-inferior to OI treatment but also resulted in the compartmentalization of iron away from the tumour. Data from this study may support the safety of iron replenishment of body iron stores with IV iron and future more comprehensive studies should be considered to confer this.

## Methods

The study was conducted in accordance with the Declaration of Helsinki (2000) of the World Medical Association with ethical approval (13/EM/0069) NRES Committee East Midlands—Nottingham 2, UK. Informed consent was obtained from all participants and all experimental protocols were approved by the University of Nottingham and the University of Wolverhampton. All methods were carried out in accordance with relevant guidelines and regulations.

### Samples

Samples for this study were obtained from the IVICA clinical trial (IVICA; IntraVenous Iron in CRC associated Anaemia) which is a multi-centre control trial that recruited anaemic adult patients with non-metastatic colorectal adenocarcinoma. Patients who provided a complete set of samples, tissue and serum, were selected for the study. Patients were randomised to receive either oral ferrous sulphate or intravenous ferric carboxymaltose for at least 2 weeks before surgery^29^. Intraoperative tissue (colorectal adenocarcinoma and paired normal tissue) were collected for the purpose of this study.

### Immunohistochemistry

Paired paraffin embedded normal and tumour tissues (n = 30/group) were dewaxed and rehydrated. Endogenous enzymes were blocked with 15% H_2_O_2_ in methanol. Antigens were retrieved by heat induction and non-specific binding was blocked using normal goat serum. Primary antibody (Supplementary Table S4) was added for overnight at 4 °C. Slides were washed with TRIS buffer and biotinylated secondary antibody was added for 30 min. The avidin–biotin complex (Vector Laboratories, UK) was used and staining was visualized using 3,3′-diaminobenzidine (Fluka, UK). Sections were counterstained with hematoxylin and mounted under coverslip. Secondary antibody alone was used as control. Images were taken on an Olympus BX51 microscope. Quantitative analysis was performed on target proteins (Supplementary Table S5) blind to the treatment. Ratio of positive cells or an analysis of immuno-reactivity with intensity scored from 0 to 2 was also determined (Supplementary Data S6). Tumours were analysed for MLH1 and MSH2 loss to determine microsatellite instability (MSI) and microsatellite stability (MSS) phenotypes. Only sections with intact tissue histology and contain normal or tumour tissues were considered for analysis.

### Perls Prussian blue staining

Tissue sections (n = 30/group) were rehydrated as per immunohistochemistry. Solution of 0.7 g ferrocyanide in 70 mL 0.5% HCl (HT20, Sigma, UK) was applied at room temperature for 60 min. Counterstain with nuclear fast red was performed for 1 min. Five high magnification fields were assessed per sample and an average score of staining was calculated. Assessors were blinded to the treatment received.

### RT-PCR

RNA was extracted from snap frozen tissue (n = 30/group) using the Thermo-Fisher Scientific mirVana™ miRNA isolation kit. Organic extraction was achieved with acid phenol chloroform and RNA purity and concentration were then determined using a NanoDrop™ 2000/2000c Spectrophotometers (ThermoFisher). Synthesis of cDNA was performed using the Qiagen^®^ QuantiTect^®^ Reverse Transcription kit. Genomic DNA was eliminated, and template RNA was mixed with reverse-transcription master-mix and incubated at 42 °C for 15 min, denatured at 95 °C and stored at − 20 °C. RT-PCR was performed for c-*MYC, IREB2, FTH1, TFRC, SLC11A2* using GAPDH control as an internal standard (Thermo Fisher Scientific) (Supplementary Table S7). TaqMan™ Gene expression master mix (Thermo Fisher Scientific) containing AmpliTaq Gold^®^ DNA Polymerase (Ultra-Pure), Uracil-DNA glycosylase, dNTPs with dUTP, ROXTM Passive Reference and optimised buffer components, was added to 100 ng of cDNA and dH2_O_ to form a 25 μL reaction mixture. Reactions without cDNA were included as negative controls. Reactions were performed in triplicate and RT-PCR was conducted using a 7500 Fast Real Time PCR System. Gene expression was normalised to GAPDH, represented as ΔCt and compared between tumour and paired normal tissues to give a ΔΔCt value. Changes in gene expression were represented a negative log of ΔΔCt and 1 regarded as normal.

### Statistics

Paired *t* test was used to test for significance between normal and tumour tissue, and between pre-treatment and post-treatment. Independent *t* test was used to compare between treatment groups. Linear regression analysis was performed compare mRNA expression and statistical significance tested with Analysis of variance. Chi square was used to analyse iron expression in tissue sections. A p-value of < 0.05 was considered significant.

## Supplementary Information

Supplementary Information.
