# Supplementary material for: Intravenous iron is non-inferior to oral iron regarding cell growth and iron metabolism in colorectal cancer associated with iron-deficiency anaemia
Source: Sci Rep. 2021 Jul 1;11:13699. doi: 10.1038/s41598-021-93155-2 (PMC8249613; doi:10.1038/s41598-021-93155-2)
Supplement: Supplementary file 1 — Supplementary Information. [file 41598_2021_93155_MOESM1_ESM.docx]

**Intravenous iron is non-inferior to oral iron regarding cell growth and iron metabolism in colorectal cancer associated with iron-deficiency anaemia.**

Hafid O. Al-Hassi*^1^**,** Oliver Ng**^*2^**, Rayko Evstatiev^3^, Manel Mangalika^4^, Natalie Worton^4^, Manuela Jambrich^3^, Vineeta Khare^3^, Oliver Phipps^1^, Barrie Keeler^1^, Christoph Gasche^3^, Austin G. Acheson**^†2^** and Matthew J. Brookes**^†1,4^**.

**Supplementary Table S1. Microsatellite instability sub-analysis of RT-PCR comparing oral versus IVI groups**

|  | Stable Mean (SD) | |  | MSI Mean (SD) | |  | P value |
| --- | --- | --- | --- | --- | --- | --- | --- |
|  | Oral (n=6) | IV  (n=7) | P | Oral  (n=1) | IV (n=2) | p |  |
| Proliferation |  |  |  |  |  |  |  |
| MYC | 3.74 (2.75) | 2.93 (1.83) | 0.628 | 18.52 (0) | 2.42 (3.1) | 0.148 | 0.115 |
| Iron transport and storage | | | | | | | |
| SLC11A2 | 1.16 (0.8) | 1.57 (1.03) | 0.445 | 3.55 (0) | 2.85 (0.29) | 0.300 | 0.009* |
| FTH1 | 0.47 (0.41) | 0.36 (0.48) | 0.635 | 0.15 (0) | 0.72 (0.14) | 0.189 | 0.665 |
| TFRC | 2.39 (1.5) | 1.28 (0.84) | 0.122 | 2.28 (0) | 2.19 (0.88) | 0.947 | 0.585 |
| Iron regulation | | | | | | | |
| IREB2 | 0.6 (0.42) | 0.5 (0.35) | 0.670 | 2.68 (0) | 1.38 (0.36) | 0.210 | 0.001* |
| ^1^ P value for tumour gene expression comparing microsatellite stable versus microsatellite instability groups | | | | | | | |

**Supplementary Table S2. Microsatellite instability sub-analysis of immunohistochemistry comparing oral versus IVI groups**

|  | Stable Mean (SD) | |  | MSI Mean (SD) | |  | P value 1 |  |  |  |  |  |  |  |
| --- | --- | --- | --- | --- | --- | --- | --- | --- | --- | --- | --- | --- | --- | --- |
|  | Oral (n=11) | IV  (n=12) | p | Oral (n=4) | IV (n=3) | p |  |  |  |  |  |  |  |  |
| Proliferation | | | | | | | |  |  |  |  |  |  |  |
| Ki67 | 0.36 (0.27) | 0.42 (0.3) | 0.550 | 0.4 (0.14) | 0.37 (0.34) | 0.712 | 0.714 |  |  |  |  |  |  |  |
| BCatM | 0.66 (0.34) | 0.66 (0.34) | 0.651 | 0.68 (0.23) | 0.6 (0.37) | 0.639 | 0.353 |  |  |  |  |  |  |  |
| BCatC | 0.57 (0.18) | 0.5 (0.18) | 0.202 | 0.55 (0.29) | 0.43 (0.11) | 0.596 | 0.562 |  |  |  |  |  |  |  |
| BCatN | 0.14 (0.18) | 0.12 (0.17) | 0.687 | 0.18 (0.37) | 0.14 (0.15) | 0.655 | 0.735 |  |  |  |  |  |  |  |
| PAK1 | 0.66 (0.09) | 0.64 (0.18) | 0.905 | 0.68 (0.13) | 0.72 (0.21) | 0.603 | 0.861 |  |  |  |  |  |  |  |
| Apoptosis and DNA damage | | | | | | | |  |  |  |  |  |  |  |
| G2HAX | 0.38 (0.31) | 0.28 (0.23) | 0.807 | 0.17 (0.13) | 0.39 (0.1) | 0.447 | 0.484 |  |  |  |  |  |  |  |
| CC3 | 0.02 (0.02) | 0.12 (0.15) | 0.243 | 0.25 (0.22) | 0.09 (0.12) | 0.224 | 0.079 |  |  |  |  |  |  |  |
| Iron transport | | | | | | | |  |  |  |  |  |  |  |
| DMT1 | 0.5 (0.19) | 0.46 (0.16) | 0.982 | 0.48 (0.2) | 0.39 (0.32) | 0.941 | 0.206 |  |  |  |  |  |  |  |
| FPN | 0.6 (0.24) | 0.63 (0.15) | 0.689 | 0.48 (0.2) | 0.39 (0.32) | 0.916 | 0.736 |  |  |  |  |  |  |  |
| TfR1 | 0.57 (0.26) | 0.67 (0.14) | 0.446 | 0.79 (0.08) | 0.45 (0.36) | 0.127 | 0.955 |  |  |  |  |  |  |  |
| ^1^ P value for tumour immunoreactivity comparing microsatellite stable versus microsatellite instability groups | | | | | | | |  |  |  |  |  |  |  |

**Supplementary Table S3. RT-PCR fold change in gene expression in oral versus IV groups**

|  | Oral  Mean fold change (SD) | IV  Mean fold change (SD) | P value^1^ |
| --- | --- | --- | --- |
| Proliferation |  |  |  |
| MYC | 5.85 (6.1) | 2.82 (1.94) | 0.070 |
| Iron transport and storage | | | |
| SLC11A2 | 1.50 (1.16) | 1.86 (1.06) | 0.974 |
| TFRC | 2.38 (1.38) | 1.49 (0.89) | 0.442 |
| FTH1 | 0.22 (0.10) | 0.43 (0.46) | <0.001* |
| Iron regulation |  |  |  |
| IREB2 | 0.90 (0.88) | 0.70 (0.51) | 0.307 |
| ^1^ P value for tumour gene expression comparing oral versus IVI groups | | | |

**Supplementary data**

**Supplementary Table S4. Immunohistochemistry antibodies**

| Target | Function | Species | Dilution | Company (Cat #) |
| --- | --- | --- | --- | --- |
| TfR1 | Iron transport | Mouse | 1:200 | Thermo scientific #13-6800 |
| DMT1 | Iron transport | Rabbit | 1:250 | Biorbyt #orb5976 |
| Ferroportin | Iron transport | Rabbit | 1:500 | Novus biologicals NBPI 21502 |
| Prussian Blue | Iron loading | N/A | N/A | Sigma-Aldrich #03899 |
| PAK-1 | Kinase phosphorylates β catenin | Rabbit | 1:50 | CST (cell signalling technology)#9664 |
| γH2AX | Histone DNA damage | Rabbit | 1:500 | CST #9718 |
| Ki67 | Proliferation | Rabbit | 1:1000 | Abcam: ab15580 |
| Cleaved caspase 3 | Apoptosis | Rabbit | 1:2000 | CST #9664 |
| P53 | Apoptosis | Rabbit | 1:500 | Santa Cruz #SC-6243 |
| Beta-catenin | Wnt signalling | Mouse | 1:300 | BD trans (BD transduction laboratories) #610153 |

**Supplementary Table 5. Immunohistochemistry semi-quantitative assessment**

| **Target** | **Localisation** | **Semi-quantitative** | **Qualitative** |
| --- | --- | --- | --- |
| TfR1 | M C a | Intensity and extent of staining* for each locale as percentage | Localisation |
| DMT1 | M C N a | Intensity and extent of staining* for each locale as percentage | Localisation |
| Ferroportin | M C b | Intensity and extent of staining* for each locale as percentage | Localisation |
| Prussian blue | C m S | Intensity and extent of staining* for each locale as percentage | Localisation |
| PAK-1 | C N | Intensity and extent of staining* for each locale as percentage | Localisation |
| γH2AX | N | Percentage of positive nuclei |  |
| Ki67 | N | Percentage of positive nuclei |  |
| Cleaved caspase 3 | N | Percentage of positive cells |  |
| p53 | N C | Percentage of positive nuclei |  |
| Β-catenin | M C N | Intensity and extent of staining* for each locale as percentage | Localisation |

M membranous, C cytoplasmic, N nuclear, b basal, a apical, m macrophages, S stroma, * Intensity of staining 0 No staining, 1 weak staining, 2 strong staining e.g. 60% strong and 40% weak cytoplasmic staining = (60 x 2) + (40 x 1) = 160 out of 200 or 80%.

**Supplementary Data S6 staining intensity**

**Weak staining**

**No staining**

**Strong staining**


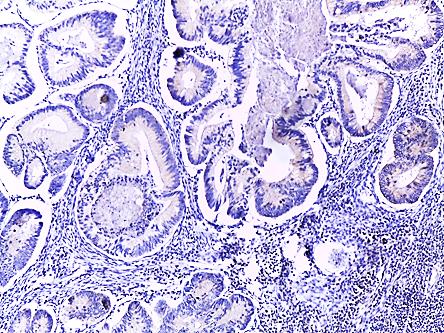

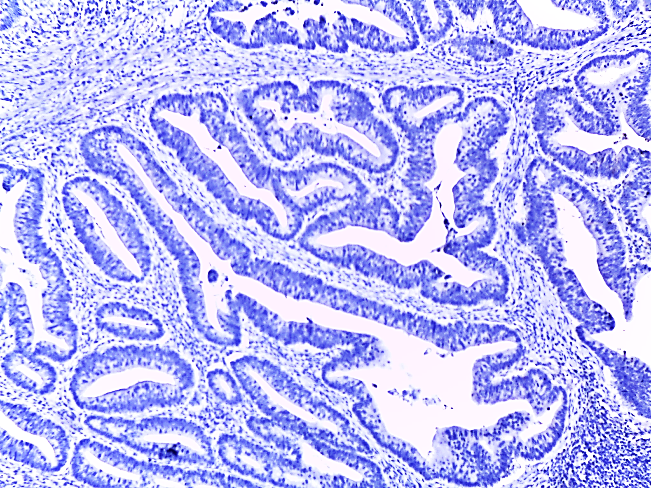

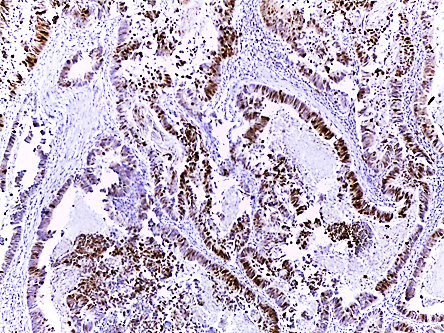


**Supplementary Table S7 RT-PCR probes**

| Gene name | Function | Assay ID |
| --- | --- | --- |
| *GAPDH* | Internal control | Hs02786624_g1 |
| *SLC11A2* | Iron transport | Hs00895685_m1 |
| *MYC* | cellular metabolism and proliferation | Hs00905030_m1 |
| *FTH1* | Iron storage | Hs01000477_g1 |
| *TFRC* | Iron transport | Hs00951084_m1 |
| *IREB2* | Iron regulation | Hs01021789_m1 |
